# The Contribution of Geogenic Particulate Matter to Lung Disease in Indigenous Children

**DOI:** 10.3390/ijerph16152636

**Published:** 2019-07-24

**Authors:** Carrington C. J. Shepherd, Holly D. Clifford, Francis Mitrou, Shannon M. Melody, Ellen J. Bennett, Fay H. Johnston, Luke D. Knibbs, Gavin Pereira, Janessa L. Pickering, Teck H. Teo, Lea-Ann S. Kirkham, Ruth B. Thornton, Anthony Kicic, Kak-Ming Ling, Zachary Alach, Matthew Lester, Peter Franklin, David Reid, Graeme R. Zosky

**Affiliations:** 1 Telethon Kids Institute, University of Western Australia, Subiaco 6008, Australia; 2 Menzies Institute for Medical Research, University of Tasmania, Hobart 7000, Australia; 3 School of Medicine, University of Tasmania, Hobart 7000, Australia; 4 School of Public Health, University of Queensland, Herston 4006, Australia; 5 School of Public Health, Curtin University, Bentley 6102, Australia; 6 School of Paediatrics and Child Health, University of Western Australia, Crawley 6009, Australia; 7 Centre for Cell Therapy and Regenerative Medicine, The University of Western Australia, Crawley 6009, Australia; 8 Department of Respiratory Medicine, Princess Margaret Hospital, Perth 6000, Australia; 9 Environmental Health Directorate, Western Australia Department of Health, Perth 6000, Australia; 10 School of Population and Global Health, The University of Western Australia, Crawley 6009, Australia; 11 Queensland Institute of Medical Research Berghofer, Brisbane 4000, Australia

**Keywords:** Indigenous, child health, geogenic, particulate matter, bacterial infection

## Abstract

Indigenous children have much higher rates of ear and lung disease than non-Indigenous children, which may be related to exposure to high levels of geogenic (earth-derived) particulate matter (PM). The aim of this study was to assess the relationship between dust levels and health in Indigenous children in Western Australia (W.A.). Data were from a population-based sample of 1077 Indigenous children living in 66 remote communities of W.A. (>2,000,000 km^2^), with information on health outcomes derived from carer reports and hospitalisation records. Associations between dust levels and health outcomes were assessed by multivariate logistic regression in a multi-level framework. We assessed the effect of exposure to community sampled PM on epithelial cell (NuLi-1) responses to non-typeable *Haemophilus influenzae* (NTHi) in vitro. High dust levels were associated with increased odds of hospitalisation for upper (OR 1.77 95% CI [1.02–3.06]) and lower (OR 1.99 95% CI [1.08–3.68]) respiratory tract infections and ear disease (OR 3.06 95% CI [1.20–7.80]). Exposure to PM enhanced NTHi adhesion and invasion of epithelial cells and impaired IL-8 production. Exposure to geogenic PM may be contributing to the poor respiratory health of disadvantaged communities in arid environments where geogenic PM levels are high.

## 1. Introduction

Indigenous communities around the world experience a significant health disadvantage [1]. Research has focused on the social determinants of this disadvantage [1,2] including loss of cultural identity [3] and low socioeconomic status [4]. However, very little attention, perhaps with the exception of household overcrowding [5] and water quality [6], has been given to modifiable exposures in the physical environment that may be contributing to poor health in Indigenous communities [1].

Indigenous children in Australia have substantially higher rates of infectious disease including respiratory [7] and ear [8] infections and are much more likely to be hospitalised for these conditions [9]. Emerging evidence suggests that environmental factors may be contributing to poor health in Indigenous communities [10]. High levels of exposure to earth-derived [geogenic] dusts is one such factor. For example, the incidence of severe respiratory infections is higher in Indigenous children living in arid communities [11], and epidemiological studies report associations between exposure to geogenic dusts and respiratory hospitalisations [12].

Geogenic dusts contain a range of pro-inflammatory compounds and bioactive metals [13]. Iron-laden particles, which are prevalent in geogenic dusts [13], are associated with an increased risk of hospitalisation for respiratory infections in children [14] and hospitalisation for respiratory conditions [15]. This is supported by our experimental studies showing that geogenic particles can cause lung inflammation [13], impair lung function [16] and exacerbate existing respiratory infections [17]. 

Our previous ecological analysis of survey data conducted in 234 remote Indigenous communities showed that high levels of dust were associated with greater community concerns regarding respiratory conditions, hearing and eyesight [18]. This study extends on this prior work [18] by using individual-level data linked to objective measures of health in order to examine the associations between reported dust levels and the prevalence of, and hospitalisation for, infectious disease in Indigenous children. We supplemented this analysis by exploring the effect of community sampled geogenic particles from these regions on in vitro cell responses to the most important pathogen for respiratory [19] and ear infections [20] in these communities—non-typeable *Haemophilus influenzae* (NTHi). 

## 2. Materials and Methods

### 2.1. Epidemiological Studies

#### 2.1.1. Data Sources

Data were from the 2000–2002 Western Australian Aboriginal Child Health Survey (WAACHS), 2003 Environmental Health Needs Survey (EHNS) and the Hospital Morbidity Data System (HMDS). 

The WAACHS was a population-representative study of Indigenous children aged 0–17 years in Western Australia. The survey used an area-based clustered multi-stage sample design. Of all eligible families, 84% consented to participate and valid information was obtained on 96% of participating children. The final sample of 5289 Indigenous children represented ~18% of all Indigenous children living in Western Australia [21].

The 2003 EHNS, run by Western Australian Government agencies, was the second comprehensive environmental health survey of discrete Indigenous communities. The survey was conducted on-site by trained Environmental Health Officers, Field Support Officers and Aboriginal Environmental Health Workers. In total, 274 communities were surveyed (covering an area >2,000,000 km^2^), with responses obtained from either a community leader or community representative and corroborated by observation where possible. 

The HMDS includes detailed information on all inpatient episodes in public and private hospitals in Western Australian. Basic demographic information along with health data are collected; admissions are coded according to the International Classification of Diseases (ICD).

#### 2.1.2. Ethics

Ethical approvals were obtained from the Western Australian Aboriginal Health Information and Ethics Committee (9-1/98; 30 January 1998) and the King Edward Memorial and Princess Margaret Hospital Ethics Committee (404/EP, 112/EP; 20 July 1999). 

#### 2.1.3. Data Linkage

The vast majority of carers of participating children in the WAACHS consented to have their WAACHS survey results linked to hospital admission records, with a successful link established in 88% of cases. HMDS data were linked by the Data Linkage Branch (DLB) of the Western Australian Government Department of Health by probabilistic record linkage [22]. EHNS data were linked using common geographic identifiers.

#### 2.1.4. Study Sample

The sample for this study included WAACHS participants where data were successfully linked to the EHNS. This included 1077 children from 66 separate communities.

#### 2.1.5. Dust Levels

Community-level dust exposure was derived from the 2003 EHNS where respondents were asked to “Rate the level of dust problems usually experienced by the community (Excessive/High/Moderate/Low/None)”. These responses were re-classified into Low/None, Moderate and High/Excessive to ensure adequate statistical power.

#### 2.1.6. Health Outcomes

In the WAACHS, carers were asked whether their children had ever been diagnosed with asthma or an ear infection, had recurring skin or chest infections, or suffered from allergies or hay fever. From the HMDS we extracted data for any respiratory condition (ICD version 10 category J), asthma (J45), upper respiratory tract infection (URTI) (J00-J06), acute lower respiratory tract infection (J12-J18 and J20-J22), influenza (J09-J11) and bronchiectasis (J47). For non-respiratory diseases, with an infection component, we extracted data for ear disease (H65-H67), eye disease (H00-H22 and H40-H42), skin conditions (L00-L08) and gastrointestinal diseases (A00-A02.0, A02.8-A05.3, A05.8-A06.2, A06.9-A09 and K52.8).

### 2.2. In Vitro Studies

#### 2.2.1. Community Sampled Dust

Geogenic PM_10_ (particulate matter < 10 µm in aerodynamic diameter) was collected and isolated using a previously established technique [13]. The sample we used in this study was collected from Karratha, Western Australia (20°44′06′′ S, 116°48′18′′ E). The chemical composition of this sample is described elsewhere [16]. This sample was chosen as it is located at approximately the midpoint of the most northern and southern communities included in this study, is in close proximity to a number of communities in the EHNS survey, and has a chemical signature, dominated by iron, aluminium and silicon, that is typical of geogenic PM in Western Australia [16].

#### 2.2.2. Cell Culture and Bacteria

NuLi-1 (ATCC CRL-4011) bronchial epithelial cells were cultured in bronchial epithelial growth medium (BEGM) consisting of BEBM basal medium and SingleQuot additives (Lonza, VIC, Monash, Australia), supplemented with 10% heat-inactivated foetal calf serum (Sigma-Aldrich, Saint Louis, MO, Australia), 0.125 µg/mL fungizone, 0.05 mg/mL gentamicin and 20 U/mL Penicillin/Streptomycin. At confluence, flasks were disrupted with 0.05% trypsin/ ethylenediaminetetraacetic acid (EDTA) (Gibco), washed in PBS and resuspended in BEGM without antibiotics. Cells were seeded (1 × 10^5^ cells) into 24-well plates and grown to confluence. For bacterial infection, we used the NTHi 86-028NP strain which readily infects epithelial cells [23].

#### 2.2.3. Cell and Bacterial Interactions in Response to PM

NuLi-1 cultures were exposed to 10 µg/mL (in BEGM) of UV-irradiated PM_10_ or iron oxide (Fe_2_O_3_; Sigma-Aldrich) as an insoluble particle control, or BEGM alone, for 24 h. Cultures were then challenged with either NTHi (multiplicity of infection [MOI] of 10:1 bacteria to cells) in PBS or PBS alone for 3 h. A gentamicin invasion assay was then used to determine NTHi attachment and cell invasion. Supernatants were collected for assessment of interleukin (IL)-6 and IL-8 levels using a bead-based immunoassay (Bio-Rad Laboratories Inc., Hercules, CA, USA). Cells were resuspended and viability assessed by staining with trypan blue and counting the cells with a haemocytometer. All experiments were conducted in triplicate and repeated at least three times on separate occasions.

#### 2.2.4. Statistical Analysis

Odds ratios and 95% confidence intervals for the association between the primary exposure (levels of dust) and the health outcomes were calculated using multivariate logistic regression models within a multi-level framework. Models were fitted taking into account the survey weights and the hierarchical structure of the data [24]. All models were adjusted for the primary exposure variable and potential confounders were selected a priori that were known to be associated with both the primary exposure and outcome: age and sex of the child (from the WAACHS; except gastrointestinal disease and ue to issues with model convergence), level of relative isolation, community population size, whether the roads in the community were sealed (from the EHNS) and whether the community had a revegetation or dust suppression program (from EHNS). Other risks were excluded if not associated with community dust exposure on the basis that we were interested in examining the total effect of dust exposure on health. Standard errors for estimates of odds ratios were calculated using a modified form of the Jack knife variance estimation technique [25]. P values were calculated using chi-squared tests adjusted for the complex sample design.

The effect of geogenic particles and NTHi infection on cell responses were assessed by ANOVA with Holm-Sidak post-hoc tests. Where appropriate, data were transformed to satisfy the assumptions of normality and homoscedasticity. Data are reported as mean (SD).

## 3. Results

### 3.1. Epidemiological Studies

#### 3.1.1. Carer-Reported Health Outcomes

Carers in communities with high/excessive community dust were more likely to report allergies/hay fever in children (OR 2.24 95% CI [1.09–4.60], *p* = 0.030) (Table 1). We found no further associations between community dust levels and the carer-reported health outcomes for asthma, recurring chest infections (and any respiratory condition), ear infections, recurring skin infections and recurring gastrointestinal infections (*p* > 0.054 in all models; Table 1).

#### 3.1.2. Hospitalisation

Children in communities with higher levels of dust were more likely to be hospitalised for any respiratory condition (moderate dust levels, OR 2.90 95% CI [1.42–5.93], *p* = 0.004; high/excessive dust levels, OR 2.56 95% CI [1.34–4.91], *p* = 0.005; Table 2), with evidence of an effect on both upper respiratory tract infections (high/excessive dust levels, OR 1.77 95% CI [1.02–3.06], *p* = 0.043) and acute lower respiratory tract infections (moderate dust levels, OR 2.04 95% CI [1.15–3.63], *p* = 0.016; high/excessive dust levels, OR 1.99 95% CI [1.08–3.68], *p* = 0.065; Table 2). Children were also more likely to be hospitalised for ear disease, but only in communities with moderate levels of dust (OR 3.06 95% CI [1.20–7.80], *p* = 0.020). There was no association between community dust levels and hospitalisation for eye disease, skin conditions or gastro-intestinal disease (*p* > 0.476 in all cases; Table 2).

The statistical models of the associations between community dust levels and hospitalisations for asthma, chronic lower respiratory disease, influenza and bronchiectasis failed to converge due to the small absolute number of cases. Accordingly, we were unable to produce robust estimates for these outcomes.

### 3.2. In Vitro Studies

#### NTHi Adhesion and Invasion

**Figure 1:**
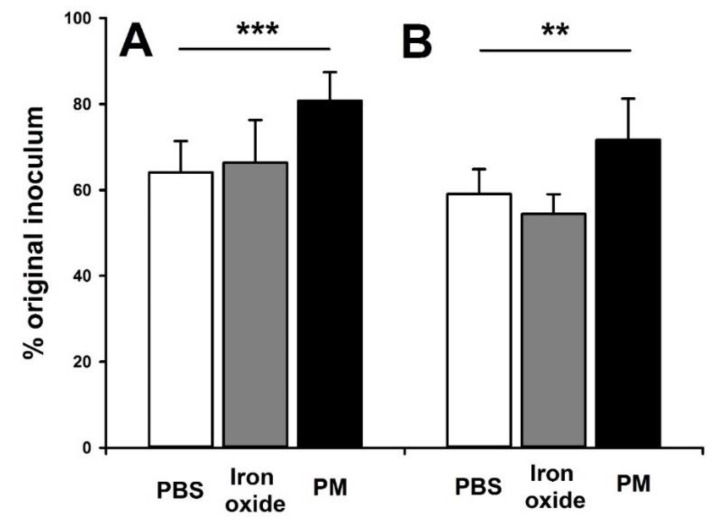
Adhesion (panel **A**) and invasion (panel **B**) of non-typeable *Haemophilus influenzae* (86-028NP strain; NTHi) in bronchial epithelial cells (NuLi-1) three hours after exposure to 10:1 MOI of the bacteria. Prior to bacterial exposure, cells were exposed to either media alone (white bars), 10 µg/mL of iron oxide particles in media (grey bars) or 10 µg/mL of community sampled particulate matter (PM)_10_ in media (black bars) for 24 h. Data are presented as mean (SD). *** indicates *p* < 0.001 and ** indicates *p* < 0.01. PM_10_ exposure enhanced both NTHi adhesion to and invasion of NuLi-1 cells while control particles (iron oxide) had no effect.

Exposure to community sampled PM_10_, but not control Fe2O3 particles (*p* > 0.17), increased both NTHi adhesion (*p* < 0.001) to the NuLi-1 cells (Figure 1A) and NTHi cell invasion (*p* = 0.002) (Figure 1B).

### 3.3. Cell Viability and Cytokine Production

**Figure 2:**
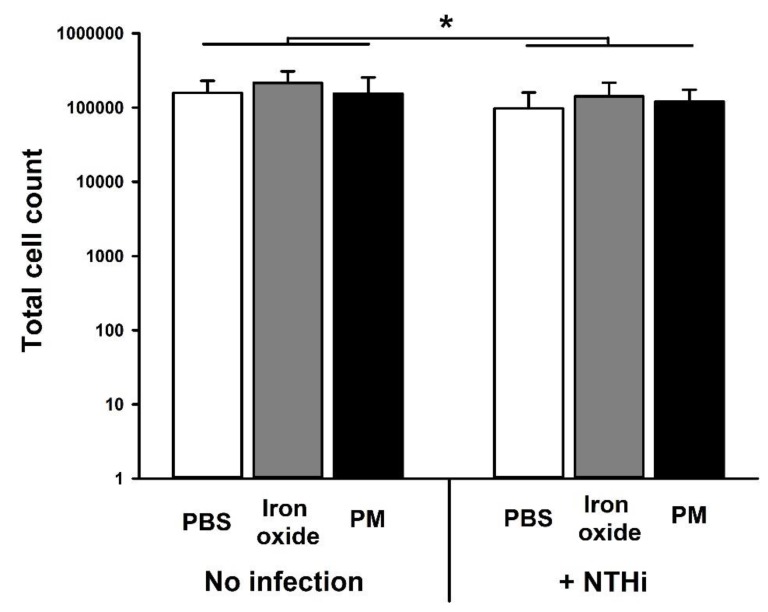
Cell viability of bronchial epithelial cells (NuLi-1) with (left) or without (right) three hours of exposure to 10:1 MOI of non-typeable *Haemophilus influenzae* (86-028NP strain; NTHi)). Prior to bacterial exposure, cells were exposed to either media alone (white bars), 10 µg/mL of iron oxide particles in media (grey bars) or 10 µg/mL of community sampled PM_10_ in media (black bars) for 24 h. Data are presented as mean (SD). * indicates *p* < 0.05. Cell viability was reduced by NTHi exposure but was not altered by particle exposure.

**Figure 3:**
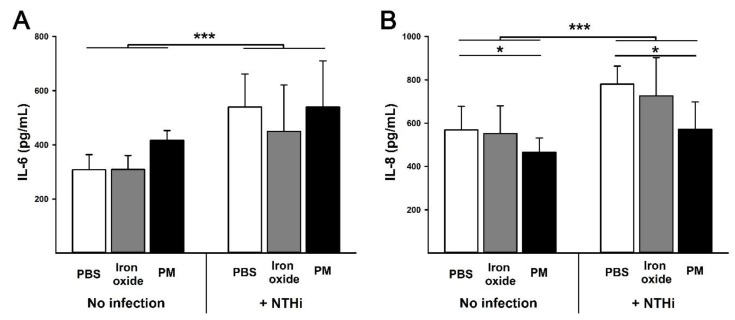
IL-6 (panel **A**) and IL-8 (panel **B**) production by bronchial epithelial cells (NuLi-1) with or without three hours of exposure to 10:1 MOI of non-typeable *Haemophilus influenzae* (86-028NP strain; NTHi)). Prior to bacterial exposure, cells were exposed to media alone (white bars), 10 µg/mL of iron oxide particles in media (grey bars) or 10 µg/mL of community sampled PM_10_ in media (black bars) for 24 h. Data are presented as mean (SD). *** indicates *p* < 0.001 and * indicates *p* < 0.05. NTHi infection increased IL-6 and IL-8 production. Particle exposure had no effect on IL-6 production. In contrast, particle exposure impaired IL-8 production by NuLi-1 cells.

There was a small, but significant (*p* = 0.02), reduction in cell viability following exposure to NTHi. However, there was no effect of PM_10_ or Fe_2_O_3_ on this response (*p* = 0.19) (Figure 2). Similarly, NTHi infection increased IL-6 production (*p* < 0.001) but this was not altered by prior PM_10_ exposure (*p* = 0.13) (Figure 3A). NTHi infection also increased IL-8 production (*p* < 0.001). However, pre-exposure to PM_10_ impaired IL-8 production (*p* = 0.01), whereas Fe_2_O_3_ particles had no effect (*p* = 0.47) (Figure 3B).

## 4. Discussion

In an ecological analysis of Indigenous communities in Western Australia, we showed that dust levels are associated with community health concerns [18]. In this study, we examined the association between community dust levels and health outcomes at the level of the individual. We found that higher levels of dust were associated with a higher prevalence of carer-reported allergies/hay fever, as well as an increased risk of hospitalisation for respiratory and ear disease in children. In follow-up in vitro studies, we found that PM_10_ increased bacterial cell adhesion and invasion and impaired IL-8 production. These observations suggest that exposure to ambient geogenic dust may be contributing to the onset and severity of diseases in Indigenous children living in remote communities.

While there were no associations between dust levels and the prevalence of infectious health conditions based on the carer-reported WAACHS data, we found associations between high dust levels and hospitalisation for respiratory conditions and ear diseases. Interestingly, the dose response for respiratory conditions appeared to plateau at moderate dust exposure levels such that there was no increase in the odds of hospitalisation for all respiratory conditions or acute lower respiratory tract infections between moderate and high/excessive dust exposure levels. We have previously shown that geogenic PM_10_, from regions in proximity to many of these communities, causes an inflammatory response in the lung [13] and subsequent impairments in lung function [16]. In follow-up studies, we showed that these particles can also independently exacerbate the response to a subsequent respiratory infection [17]. Based on current evidence, this exacerbation appears to be due to the suppression of the pathogen clearance response [17].

The relationship between dust exposure and hospitalisation for ear disease was more complex and somewhat counter-intuitive; moderate levels of dust were associated with increased odds of hospitalisation for ear disease whereas high/excessive dust levels were not. We have no explanation for this observation. If we accept that there is indeed an association between dust exposure and ear disease, it may be explained by the overlap in pathogens that regularly infect the airways and middle ear of Indigenous children. For example, non-typeable *Haemophilus influenzae* (NTHi) is associated with both chronic, severe respiratory disease [19] and chronic ear disease [20].

In vitro we found that PM_10_ enhanced NTHi adhesion and invasion in epithelial cell cultures. Adhesion of NTHi to epithelial cells is critical for the establishment of persistent infection [26] and invasion of epithelial cells allows the bacteria to avoid eradication by the immune system [27]. While PM_10_ exposure had no impact on cell viability or IL-6 production, it did impair IL-8 production both at baseline and in response to NTHi infection. IL-8 is a critical cytokine in the response to NTHi infection and is a potent neutrophil chemoattractant [28]. The early recruitment of neutrophils to the site of NTHi infection is a critical determinant of bacterial clearance [29]. This effect, combined with the increased bacterial adhesion and invasion we observed, means that exposure to geogenic PM_10_ is likely to increase the severity of NTHi infection. The absence of an IL-6 response is interesting to note given other studies have shown increased production of this cytokine using similar particles [30]. It is possible that this is due to the cell line dependence of the cytokine response [30]. Thus, further studies are required to fully elucidate the potential impact of these particles on lung epithelial cells and other cells involved in the innate response to inhaled PM_10_.

Increased levels of dust were also associated with an increased odds of carer-reported allergies/hay fever. This suggests that higher levels of exposure to dust in remote communities increase the risk of allergic sensitisation in children. The physico-chemical composition of dust in remote communities, while dominated by geogenic particles, is likely to be complex and will include a range of particulate allergens (e.g., pollen). These allergens may exist as particles, or adhere to other particulate matter [31], which then facilitate access to the mucosal surfaces. In this instance we do not know whether the association we observed is driven by increased allergen exposure or higher particulate loads causing an adjuvant effect [31]. Also, we do not know whether there is an increase in hay fever, allergy or both and what “type” of allergy (e.g., eczema, hay fever, etc.) is driving the association. Notwithstanding these limitations, our data suggest that higher dust levels increase the risk of allergies/hay fever in Indigenous children living in remote communities. Interestingly, there were no associations for other carer-reported health conditions which may suggest that dust levels have low importance in driving the high prevalence of infectious disease in Indigenous children but do have an impact on disease severity (hospitalisation). However, it is important to consider the high background rate of disease in these communities which may “normalise” the perception of illness making the carer less likely to report the presence of a disease that would otherwise be diagnosed by a physician.

Collectively, our data support a role for ambient geogenic PM_10_ in contributing to the prevalence and severity of respiratory and ear disease in Indigenous children living in remote communities; although, it should be acknowledged that there are a few limitations. The first is the use of self-reported dust levels for the community as the exposure measure. However, one of the strengths of our study was the large number of communities that contributed data to our analysis (66 in total); this allowed us to link the dust exposure data (from the EHNS) with the WAACHS and HDMS. This would not have been feasible due to the low population numbers in the communities (median 120; interquartile range [10–60]) and the area covered by the study (>2,000,000 km^2^).

A second limitation relates to the temporal issues in our study design. The study participants were drawn from a cross-sectional survey in 2000–2002, with risk factors established from the 2003 EHNS. While these data sources are several years old, we know that environmental conditions in remote Indigenous communities have been relatively stable over time. For example, 31% of the Australia Indigenous population living in remote communities lived in overcrowded housing conditions in 2004, compared to 27% in 2014, and only 15.3% of communities were connected to a town water supply in 2001, compared to 17.6% in 2006 [32]. Accordingly, the data continue to provide a robust assessment of the environmental and social conditions of Indigenous children living in discrete communities today [32]. This also mitigates the potential problems associated with the slight temporal difference between the exposure outcomes (2003) and the health outcomes (2000–2002). Further, it should be noted that our measure of exposure is a snapshot in time of a general perception by a community representative and we acknowledge the potential for reporting bias, whereby communities that have a high rate of respiratory diseases may be more likely to report high dust levels. It is also worth noting that other environmental factors in some communities that we did not capture, such as thermal stress, may be making an important contribution to respiratory morbidity [33], although there was no evidence of spatial variations in our data that would suggest a link between the health outcomes and average ambient temperature.

## 5. Conclusions

In summary, we have shown that higher levels of reported dust are associated with an increased risk of hospitalisation for respiratory conditions and ear diseases and an increased prevalence of allergies/hay fever in Indigenous children. We were also able to establish a biologically plausible link between these exposures and the health outcomes we observed in vitro. As the evidence for this association grows, it is important to reflect on the potential contribution of environmental conditions to the health disparity between Indigenous and non-Indigenous children, particularly in the case of ambient dust levels, which can be easily reduced by simple remediation measures such as dust suppression or revegetation programs [18]. This work has important global health ramifications for communities in arid environments where geogenic PM_10_ levels are high, particularly those experiencing significant social disadvantage.

## Figures and Tables

**Table 1:** Relationships between perceived level of dust in the community (Environmental Health Needs Survey (EHNS)) and parent-reported (Western Australian Aboriginal Child Health Survey (WAACHS)) health outcomes among Indigenous children aged 0–17 years in discrete communities of Western Australia, 2000–2002 ^a^.

Dust Level	OR	95% CI	*p*-value ^c^
***Asthma***			
Low/None	1.00	..	..
Moderate	0.68	0.29–1.59	0.375
High/Excessive	1.40	0.63–3.12	0.411
***Any respiratory condition*** **^b^**			
Low/None	1.00	..	..
Moderate	0.89	0.49–1.64	0.719
High/Excessive	1.37	0.87–2.17	0.178
***Recurring chest infections***			
Low/None	1.00	..	..
Moderate	0.60	0.19–1.86	0.372
High/Excessive	1.05	0.43–2.53	0.920
***Ear infections***			
Low/None	1.00	..	..
Moderate	0.73	0.41–1·28	0.269
High/Excessive	0.60	0.36–1·01	0.054
***Recurring skin conditions***			
Low/None	1.00	..	..
Moderate	1.00	0.43–2.32	0.995
High/Excessive	1.37	0.58–3.24	0.477
***Allergies and hay fever***			
Low/None	1.00	..	..
Moderate	1.08	0.46–2.52	0.865
High/Excessive	2.24	1.09–4.60	0.030
***Recurring gastrointestinal infections***			
Low/None	1.00	..	..
Moderate	1.44	0.47–4.40	0.520
High/Excessive	1.11	0.48–2.56	0.814

^a^ Results are derived from multivariate logistic regression models using a multi-level framework. All models are adjusted for age, sex, level of relative isolation, community population size, whether the roads were sealed, and whether the community had a revegetation program. Each wafer (health variable) represents a separate model. ^b^ Includes asthma (ever), recurring chest infections, wheezing (ever), and taken medication for asthma/wheezing (last 12 months). ^c^ Calculated using chi-square tests adjusted for the complex sample design.

**Table 2:** Relationships between the perceived level of dust (EHNS) in the community and selected health outcomes (HMDS) among Indigenous children aged 0–17 years in discrete communities of Western Australia, 2000–2002 ^a^.

Dust Level	OR	95% CI	*p*-value ^d^
***Any respiratory condition*** **^b^**			
Low/None	1.00	..	..
Moderate	2.90	1.42–5.93	0.004
High/Excessive	2·56	1.34–4.91	0.005
***Selected ear diseases*** **^c^**			
Low/None	1.00	..	..
Moderate	3.06	1.20–7.80	0.020
High/Excessive	1.90	0.88–4.13	0.105
***Selected eye diseases*** **^e^**			
Low/None	1.00		
Moderate	1.37	0.43–4.36	0.598
High/Excessive	1.20	0.42–3.41	0.734
***Selected skin conditions*** **^f^**			
Low/None	1.00	..	..
Moderate	1.13	0.63–2.02	0.673
High/Excessive	0.99	0.61–1.62	0.976
***Selected gastrointestinal diseases*** **^g^**			
Low/None	1.00	..	..
Moderate	0.79	0.42–1.50	0.476
High/Excessive	1.01	0.61–1.66	0.971
***Upper respiratory tract infections*** **^h^**			
Low/None	1.00	..	..
Moderate	1.73	0.77–3.91	0.188
High/Excessive	1.77	1.02–3.06	0.043
***Acute lower respiratory tract infection*** **^i^**			
Low/None	1.00	..	..
Moderate	2.04	1.15–3.63	0.016
High/Excessive	1.99	1.08–3.68	0.028

^a^ Results are derived from multivariate logistic regression models using a multi-level framework. All models are adjusted for age and sex (except the model for gastrointestinal diseases and URTIs), level of relative isolation, community population size, whether the roads were sealed, and whether the community had a revegetation program. Each wafer (health variable) represents a separate model. ^b^ Defined as all International Classification of Diseases (ICD)-10 codes under category J. ^c^ Defined as ICD-10 codes under categories H65-H67. ^d^ Calculated using chi-square tests adjusted for the complex sample design. ^e^ Defined as ICD-10 codes H00-H22 and H40-42. ^f^ Defined as ICD-10 codes L00-08. ^g^ Defined as ICD-10 codes A00-A02.0, A02.8-A05.3, A05.8-A06.2, A06.9-A09 and K52.8. ^h^ Defined as ICD-10 codes J00-J06. ^i^ Defined as ICD-10 codes J12-J18 and J20-J22.
